# Validation and User Evaluation of a Sensor-Based Method for Detecting Mobility-Related Activities in Older Adults

**DOI:** 10.1371/journal.pone.0137668

**Published:** 2015-09-11

**Authors:** Hilde A. E. Geraedts, Wiebren Zijlstra, Helco G. Van Keeken, Wei Zhang, Martin Stevens

**Affiliations:** 1 University of Groningen, University Medical Center Groningen, Center for Human Movement Sciences, Groningen, The Netherlands; 2 Institute of Movement and Sport Gerontology, German Sport University Cologne, Cologne, Germany; 3 Philips Research Europe, Eindhoven, The Netherlands; 4 University of Groningen, University Medical Center Groningen, Department of Orthopaedics, Groningen, The Netherlands; Texas Tech University Health Science Centers, UNITED STATES

## Abstract

Regular physical activity is essential for older adults to stay healthy and independent. However, daily physical activity is generally low among older adults and mainly consists of activities such as standing and shuffling around indoors. Accurate measurement of this low-energy expenditure daily physical activity is crucial for stimulation of activity. The objective of this study was to assess the validity of a necklace-worn sensor-based method for detecting time-on-legs and daily life mobility related postures in older adults. In addition user opinion about the practical use of the sensor was evaluated. Twenty frail and non-frail older adults performed a standardized and free movement protocol in their own home. Results of the sensor-based method were compared to video observation. Sensitivity, specificity and overall agreement of sensor outcomes compared to video observation were calculated. Mobility was assessed based on time-on-legs. Further assessment included the categories standing, sitting, walking and lying. Time-on-legs based sensitivity, specificity and percentage agreement were good to excellent and comparable to laboratory outcomes in other studies. Category-based sensitivity, specificity and overall agreement were moderate to excellent. The necklace-worn sensor is considered an acceptable valid instrument for assessing home-based physical activity based upon time-on-legs in frail and non-frail older adults, but category-based assessment of gait and postures could be further developed.

## Introduction

Regular physical activity is crucial in the prevention of health decline in older adults [1]. However, older adults generally do not engage in sufficient daily physical activity [2]. Six to ten percent of older adults aged 70 and over in the United States engage in the required minimum of 30 minutes of moderate activity five days a week [3,4]. In The Netherlands only ten percent of seniors are sufficiently active regarding these guidelines [2]. World wide numbers vary between 2.4 and eighty-three percent for older adults 60 years and over, depending on the definition and measurement method used [5].

Accurate measurement of daily physical activity is crucial for assessment and stimulation of activity. Many daily activity measurements are based upon self-report, such as the Physical Activity Scale for the Elderly (PASE) [6] and the International Physical Activity Questionnaire (IPAQ) [7,8]. However, self-report measures are generally biased due to recall bias and social desirability [9].

Step counters and accelerometers measure daily physical activity objectively and are therefore less prone to bias in measurement [10,11]. Though easy to use, step counters do not provide an accurate measurement of daily physical activity. Current developments in technology enable more accurate objective measurement of physical activity by means of accelerometers. These small, lightweight body-worn sensors can provide an unobtrusive method to measure subjects’ physical activity for longer periods in non-laboratory environments and daily life [12]. Body-worn sensors have been demonstrated to have an accuracy in detecting gait or postures in healthy and physically impaired older adults varying between 64.4 to 100.0 percent under standardized laboratory circumstances [13–20]. When assessing accuracy under semi-standardized or real life conditions, accuracy is lower [15, 16]. Real life measurement of gait and postures, in order to assess daily physical activity, could still be improved. A recent development in body-worn sensors is a necklace-worn motion sensor which may be used to measure physical activity by detecting and monitoring postures and walking. This sensor is especially suitable for assessment of daily activity due to its necklace-worn design. A sensor that is worn in a well-known fashion, such as as a necklace or around the wrist, is considered least intrusive by wearers and therefore more suitable for daily wearing [21]. This is a major advantage when compared to earlier body-worn sensor methods, that were mainly placed on the hip lower back, or had multiple sensor attachments [13–20]. The necklace-worn sensor is a miniature hybrid sensor which contains a 3D-MEMS accelerometer together with a barometric pressure sensor. The sensor assesses “time-on-legs” (ToL: the time spent actively on the legs, i.e. standing, shuffling around, walking and transitions in between). ToL provides a novel, very suitable measurement of daily activity in older adults, since in (frail) older adults an important part of their daily activity consists of activities such as standing and shuffling around within their own home. In general, only more vigorous activities (such as for instance outdoor walking, cycling) are considered physical activity in objective activity measurement and used for performance tests indicating subject’s progress [22]. However, in order to depict (frail) older adults’ activity and detect changes in their daily activity caused by physical activity interventions, the indoor less vigorous activity should also be included in physical activity assessment. The sensor-based method for activity detection proposed in this paper takes into account also this indoor light activity and should therefore be appropriate for detection of daily life mobility related postures and activity in (frail) older adults.

Primary objective of this study was to assess the validity of a sensor-based method to detect time-on-legs and daily life mobility related postures in older adults based on a necklace-worn motion sensor. Secondary objective was to evaluate user opinion about the practical use of the sensor.

## Materials and Methods

### Design

This study consisted of validation of a sensor-based method for activity detection in the home environment and evaluation of user opinion. The study protocol was approved by the Medical Ethical Committee of University Medical Center Groningen (METc 2011/022).

### Subjects

Subjects were frail and non-frail older adults. Subjects were community-dwelling or living in an older adult home, aged ≥70 years and able to walk 10 metres without support or with a cane or walker. Frailty was assessed by means of the Groningen Frailty Indicator (GFI). Frailty is defined as “the state of vulnerability to stressors that is independent of any specific disease or disability but that is common in older people and predisposes them to various adverse health outcomes” [23]. The GFI is a 15-item screening instrument for the level of frailty, stating questions on physical, cognitive, social, and psychological characteristics [24]. All scores are dichotomized, a score of 1 indicating a problem or dependency. GFI scores reign from 0 to 15, 0 indicating no debilitations in functioning and 15 indicating major problems in physical, cognitive, social and psychological functioning [24]. Subjects with a total score ≥4 are considered frail.

Exclusion criteria were orthopedic impairments that debilitate the ability to walk unsupported for ten metres, total hip- or knee replacement surgery in the previous six months, having had a stroke within the last six months, Parkinson’s disease stage 4/5 or other neurologic diseases that can impair daily functioning or visual problems to a degree that make it impossible for the subject to accurately read the questionnaires or walk around safely.

Subjects were recruited from an existing list of older adults that participated in earlier studies and using flyers and information gatherings in neighborhoods where many older adults live or at residential homes in the city of Groningen, the Netherlands. Written informed consent was obtained before start of the measurements.

### Sensor signal processing and classification

The miniature hybrid sensor contains a 3D-MEMS accelerometer and a barometric pressure sensor, and is worn as a necklace. Accelerometry data were sampled at 50 Hz with a range of 4g, barometric data were sampled at 25Hz. A micro-SD card was used for storage and exchange of data. The weight of the sensor was about 30 grams and it measured 55 by 25 by 10mm (Philips Research, Eindhoven). An algorithm to classify periods that a person is on his/her legs—time-on-legs (ToL) was developed. The algorithm aimed to detect periods a person was active on his/her legs. Details of sensor signal processing and feature computation are described in Zhang et al. [25]. We briefly describe the classification of ToL in the rest of this section.

A low-pass filter was applied to the raw 3D-acceleration and the air pressure signal. De-noised and smoothed signals were the input to each of the movement and posture detection modules to detect ToL related activities: 1). active period; 2). sit/stand transfer; 3). walking and 4). lying. The outputs of the aforementioned modules were then fed to a heuristic classifier to detect ToL.—Active period: active and inactive periods were determined by the signal intensity. An experimental threshold, based on representative pilot data (separate from the data reported upon in this manuscript), was applied to categorize signal bouts with sustained intensity above the threshold to active periods.—Sit/stand transfer: features including cross correlation with transfer template signal, time difference between signal peak and valley, sensor orientation, signal intensity before or after a transfer and altitude change during the transfer were computed and fed into Support Vector Machine (SVM). The SVM then determined whether the signal bout represented a sit-to-stand transfer or a stand-to-sit transfer.—Walking: repetitive peaks present in the norm of 3D acceleration signals were selected. Number of peaks in the signal, peak heights and peak intervals in one bout were feature vector to a threshold-based classifier to detect walking steps.—Lying: sensor orientation was first computed to decide the position of z-axis. A sustained period of time showing z-axis of the sensor in (close) perpendicular position to the horizontal plane was classified as lying period.—Heuristic classifier: outputs of the above mentioned movement and posture classification modules were first assembled into one output signal with a label of movement or postures second-by-second. Signals which received multiple labels from different modules were corrected following a descending priority of sit/stand transfers, walking and lying. For example, a signal bout labeled with sit-to-stand transfer and walking was corrected as sit-to-stand transfer since the transfers had higher priority than walking. In addition, active and inactive periods were corrected to the 1). label of standing if the signals succeeding a sit-to-stand transfer or walking and preceding a stand-to-sit transfer or walking; or to the 2). label of sitting if the signals succeeding a stand-to-sit transfer and preceding a sit-to-stand transfer or lying. Finally, signals classified as sit/stand transfers, walking, standing and the un-labeled active periods were categorized to ToL [25].

Training of the algorithm and its thresholds for detection was performed on separate but representative pilot data incorporating healthy adult subjects as well as frail and non-frail older adults. These data included sit-to-stand, lying and walking exercises as well as the protocol as presented in the manuscript. These data were only used for education and testing of the algorithm, and not included in the data reported in this paper.

### Video validation

Validation of the sensor-based method included a standardized movement protocol as well as a free movement protocol. The standardized movement protocol included standing up, walking, lying and sitting while wearing the sensor and being filmed. Also, the Timed Up and Go Test (TUG) [26, 27] and the Five Times Chair Rise Test were included. Participants were allowed to take rest in-between exercises or skip exercises that were too difficult. Circumstances were standardized as much as possible by removing possible distractions and subjects were only addressed for instructing or helping them when performing the protocol. The protocol is shown in Table 1.

**Table 1:** Standardized protocol.

	Activity
**1**	Standing still in front of the chair for 5 seconds
**2**	Sit down on the chair
**3**	Timed Up and Go- slow condition
**4**	Timed Up and Go- normal condition (3 repetitions)
**5**	Timed Up and Go- fast condition
**6**	Five Times Chair Rise
**7**	Standing up from the chair (STS)
**8**	Bending over to pick up pen from the floor*
**9**	Lying down on bed- on back, turn on right side and stand up again
**10**	Lie down on the floor and get up again*
**11**	Walking 10m using cane or walker**

* These activities are optional

** Walking 10m using cane or walker only for subjects that use these in daily life

The free movement protocol consisted of 30 minutes of self-chosen activities such as performing household chores while being filmed. Subjects were instructed to perform household chores or indoor leisure activities at will, and were provided with suggestions when they could not think of any themselves. Common activities chosen were vacuuming, reading, preparing tea or coffee, cleaning dishes and watering plants. The videos from the standardized movement protocol and the free movement protocol were annotated in a video analysis program, Noldus “The Observer” version 10.5 (Noldus Information Technology, The Netherlands). Scoring observations included start and end of each pre-defined activity, namely sitting, standing, walking and lying. The performance of the activities as observed by video was taken as the gold standard. The video camera was kept perpendicular to the actions of the participant whenever possible, which resulted in a side view of all movements. Scoring was performed by three independent extensively trained assistants. All videos were rated by two of the three raters, which were assigned randomly to the videos.

### User evaluation

User evaluation was based on a week of wearing the sensor in daily life after the initial visit, by means of a user evaluation questionnaire. Participants were instructed to wear the sensor day and night, but if wearing the sensor while sleeping was uncomfortable they were allowed to leave off the sensor during the night. At the end of the week, a researcher administered the user evaluation questionnaire about the sensor. The user evaluation questionnaire consisted of seven statements addressing comfort, weight, size and usability of the sensor which had to be scored between 1 and 5 (1 meaning “Do not agree at all” and 5 meaning “Completely agree”), and an optional additional question regarding suggestions for improvement of the sensor. A high mean score on the questionnaire indicates a positive opinion on wearing the sensor. The additional question regarding improvements for the sensor was assessed separately.

### Data analysis

Percentage of agreement was calculated for assessment of inter-rater reliability on the video annotation. Inter-rater reliability (Intra-class Correlation Coefficient, Two way Random, average measures) was deemed sufficient when the Intra-class Correlation Coefficient (ICC) exceeded 0.8 [28].

Physical activity was assessed based on “time-on-legs” (ToL), which algorithm was described into more detail in section 2.3. In addition, a category-based analysis of gait and postures was performed for more in-depth information on gait and posture detection. The categories were lying, sitting, standing and walking. Lying was defined when the person’s trunk was in a horizontal position with the back, stomach or side touching a horizontal underground without signs of further movement. Sitting was defined when the person’s trunk was in a vertical seated position without movement in the trunk. The angle between the legs and the trunk should be about 90 degrees. Standing was defined when the person was in an upright vertical position with no or only a small displacement, but no distinctive steps, of the feet [28,29]. Walking was defined when the person was moving the feet forward in a walking pattern with the trunk in a forward displacement, from when the heel of the foot cleared the ground for the initial step until the foot of the closing step made complete contact with the floor, with a minimum of 2 steps [29, 30]. For data-analysis purposes, all activities were number-coded representing the corresponding activity category.

For validity calculations, the percentage of correspondence of the sensor outcomes and the observational outcomes was calculated over activity data of all separate subjects. Afterward, sensitivity, specificity and overall agreement measures were calculated group-wise based on second-by-second analysis. For example, the definitions used for the calculations for “walking” were as follows [29, 30]:

1. Sensitivity: (total duration that the video observation and the sensor corresponded at the same moment for walking/total duration that walking was observed on video) 100%
2. Specificity: “(total duration that the video observation and the sensor corresponded at the same moment for detected non-walking activities/total duration of non-walking activities as observed on video) 100%”

Afterwards, overall agreement on all categories was calculated as follows [29, 30]:

3 Overall agreement: (total duration that the video observation and the sensor corresponded at the same moment for all categories/total duration that the activities were observed on video) 100%

Sensitivity, specificity and overall agreement were calculated ToL-based as well as category-based. Cut-off values for sensitivity, specificity and overall agreement were defined as: ≤ 40% insufficient, 40%> till ≤ 60% moderate, 60% > till ≤ 80% good and > 80% excellent agreement [28, 31], as defined in Fleiss et al [31]. All measures were also calculated for frail and non-frail subjects separately. An independent samples T-test was used to assess whether sensor performance in both physically differently performing groups was comparable.

Statistical analyses were performed in Matlab 2012a, Microsoft Office Excel 2010 and SPSS version 16.0.

## Results

Twenty subjects were included in the study (4 male, 16 female, mean age ± SD 78.7 ± 5.4). Seven were considered frail, thirteen non-frail. Mean GFI score was 2.7 ± 2.1. Table 2 summarizes the subjects’ characteristics.

**Table 2:** Subject characteristics.

Characteristics	Total group (n = 20)	Frail subjects (n = 7)	Non-frail subjects (n = 13)
**Age (year)**	79.7 ± 5.7	84.1 ± 2.6	77.3 ± 5.5
**Gender (male/female)**	5/15	0/7	5/8
**Height (cm)**	165.0 ± 0.1	161.1 ± 0.1	166.5 ± 0.1
**Weight (kg)**	79.6 ± 11.2	79.7 ± 11.3	79.6 ± 11.5
**GFI score Living conditions (independent/dependent) User of cane or walker(yes/no)**	2.7 ± 2.1 16/4 9/11	6.0 ± 2.6 4/3 7/0	1.4 ± 1.1 13/0 2/11

Values are means ± standard deviation

The ICC for inter-rater agreement of the video observation based upon category-wise assessment was 0.97 in the standardized assessment and 0.91 in the free movement protocol. The video observation was therefore deemed sufficient to be used as a reference method [28].

### Validity

#### Standardized protocol

For the video validation of the sensor in the standardized assessment, 11285 seconds of data (3.13 hours) were collected. Mean duration per subject was 564.25 seconds.

Table 3 shows the average correspondence of video observation and sensor data in the standardized assessment. Time-on-leg based, sensitivity, specificity and overall agreement were good to excellent. Category-based, sensitivity, specificity and overall agreement measures varied from good to excellent with the exception of lying in which the sensitivity was insufficient. There were no significant differences between frail and non-frail older adults (p ≥ 0.61).

**Table 3:** Mean Sensitivity, Specificity and Percentage Agreement Standardized Assessment Protocol.

SMM	Sensitivity			Specificity			Overall Agreement		
Video	Overall (n = 20)	Non-Frail (n = 13)	Frail (n = 7)	Overall (n = 20)	Non-Frail (n = 13)	Frail (n = 7)	Overall (n = 20)	Non-Frail (n = 13)	Frail (n = 7)
**TOL**	**72.1**	**70.2**	**75.7**	**87.7**	**85.8**	**91.1**	**87.5**	**79.2**	**86.0**
**Sitting**	82.3	79.2	88.1	65.8	67.2	63.2	74.9	72.9	78.8
**Standing**	61.5	61.3	61.8	83.1	80.7	87.4	78.4	75.9	83.0
**Walking**	61.0	63.6	56.3	97.7	97.5	98.4	93.1	93.3	92.6
**Lying**	31.8	35.6	26.2	99.5	99.3	99.7	97.2	96.9	97.5

#### Free movement protocol

For the video validation in the free movement protocol, in total 35855 seconds of data (9.96 hours) were collected. Mean duration was 1708.95 seconds.

Table 4 shows the average correspondence of video observation and sensor data in the free movement protocol. Time-on-leg based, sensitivity, specificity and overall agreement were excellent. Category-based sensitivity, specificity and overall agreement measures were moderate to excellent with the exception of walking in which the sensitivity was insufficient. There were no significant differences between frail and non-frail older adults (p ≥ 0.07).

**Table 4:** Mean Sensitivity, Specificity and Percentage Agreement Free Movement Protocol.

SMM	Sensitivity			Specificity			Overall Agreement		
Video	Overall (n = 20)	Non-Frail (n = 13)	Frail (n = 7)	Overall (n = 20)	Non-Frail (n = 13)	Frail (n = 7)	Overall (n = 20)	Non-Frail (n = 13)	Frail (n = 7)
**TOL**	**84.3**	**83.2**	**86.2**	**94.2**	**92.2**	**97.7**	**81.6**	**85.0**	**91.6**
**Sitting**	84.2	83.6	85.2	83.0	84.7	79.9	84.4	84.6	85.1
**Standing**	77.5	77.7	77.2	68.6	65.3	74.7	73.1	70.7	77.4
**Walking**	42.9	48.1	33.5	96.7	95.7	98.6	87.9	86.2	90.9
**Lying**	57.6	62.6	32.5	99.9	99.8	99.9	99.7	99.5	99.9

#### User evaluation

For user evaluation, 142 days of daily life data were collected (mean per subject seven days). All subjects wore the sensor during daytime of all requested days. Sixteen subjects wore the sensor while sleeping. The average score on the user evaluation questionnaire was 4.4 (SD ± 0.6; range 2.4–5.0) on a scale of 1 to 5. Recommendations regarding improvements mainly concerned the sensor’s shape: five subjects indicated that it would be a possible amelioration for the sensor to be thinner.

## Discussion

In order to be able to measure and provide feedback on daily activity in real life, it is imperative that activity assessment of a sensor-based method is accurate. Overall, the proposed sensor-based method in this paper provided an excellent estimation of ToL, and a moderate to good estimation of gait and postures in the home situation in standardized as well as free movement conditions. User acceptance is high. The sensor is deemed suitable for daily activity assessment in real life in (frail) older adults.

Accuracy of sensor-based methods to measure physical activity, gait and postures based upon accelerometry have shown a high validity under laboratory circumstances with sensitivity and specificity reaching above 0.95 and overall accuracy ≥ 87% [14–16,30,31]. Sensitivity and specificity are however generally lower in the home environment than under laboratory circumstances [32], due to for instance the lack of standardization of movement instructions. However, the current sensor-based method shows equally excellent accuracy in validation in the home environment based on ToL detection, comparable to outcomes of other sensor-based measures under laboratory circumstances [14–16,32,33].

Accuracy of ToL detection in the free movement protocol was comparable to the standardized protocol, contrary to what one would expect due to the more unpredictable nature of free movement as opposed to standardized circumstances. The standardized assessment involved many transitions within a short time span while in free movement, many older adults chose several longer walking, sitting- or standing periods. With fewer transitions to detect, agreement of ToL detection is higher in free movement. This is promising for accuracy of the sensor-based method in daily life, since the free movement is designed to resemble daily life situations [16].

ToL assesses the daily activity from a macro perspective, which might be a useful and relevant performance indicator for the older population. A further analysis into specific movement and posture categories was conducted to gain additional information of the daily activity from a micro perspective. We analyzed the following four categories: Walking, Sitting, Standing and Lying, which were also studied in other literature. With detection of sitting and standing comparable to or better than earlier results in literature under laboratory circumstances [16,29, 30], detection of lying proves to be the most difficult to detect (sensitivity 0.32) even though specificity of detection of lying was excellent (0.99). This difficulty in detection is most probably due to the short lying intervals in the data. Due to the short durations small errors in synchronisation had a large negative impact on the accuracy of detection. A shift of merely one second is a large error in detection in a two-second lying interval and results in low sensitivity. However, when addressing the number of lying intervals accurately detected the sensor detected fifteen out of sixteen intervals in the free movement and 15 out of 26 intervals in the standardized assessment. The missed intervals were mostly very short lying bouts (2–9 seconds), including several seconds of “turning over onto the side” halfway during the interval. Since in real life lying mostly is prominent during long periods of resting and sleeping, the current protocol is most probably not representative for the detection of lying in real life. Detection during the longer periods of lying in daily life is expected to be higher based on these results. Also, a large part of the inaccuracy in lying is caused by the design of the sensor as a necklace. When lying down the sensor often slides to the side, causing additional noise in the detection signal. Especially in the aforementioned short lying bouts of 2–9 seconds, this sliding down comprises a substantial part of the lying bout and therefore causes a large inaccuracy. In addition, walking provided some challenges causing a moderate sensitivity. These challenges were mostly due to low sensitivity to detection of walking periods in frail subjects. Frail older persons generally walk slower, more inconsecutive and have more body sway during walking, which may disturb detection of walking [34, 35]. Also, all frail subjects used a cane or walker. Cane use is of large influence on gait pattern, introducing asymmetry and weight shift in the walking pattern compared to walking without cane use [35]. The gait patterns and asymmetry due to cane use can hamper recognition of the walking pattern by the necklace-worn sensor, which may explain the slightly lower sensitivity to walking of the sensor in frail older adults. Overall, regardless of the challenges provided by lying and walking, hampering it’s use for specific gait- and posture detection in the home situation, the sensor excellently detected ToL and is therefore able to make an accurate distinction between time spent active and time spent inactive, which is crucial for accurate daily activity detection.

Next to the accuracy of detection algorithms, the use of body-worn sensors should be unobtrusive and designed not to hamper wearers during everyday life. If a sensor is not comfortable to wear or difficult to use, subjects will reject it [36] and adherence will be low. Recent studies provide that people prefer body-worn sensors on the wrist, torso, arm or waist [37]. Acceptability is heightened when the sensor is worn around the wrist, or as a necklace. The high adherence in wearing and score in the user evaluation in this study emphasize these findings.

### Strengths and limitations

In previous literature, sensor-based methods for measurement of daily physical activity have mostly been tested under laboratory circumstances [15]. However, assessment under laboratory circumstances is not applicable to daily life [35]. In the current study, validation of the movement registration method for older adults is performed in a semi-structured way including standardized assessment, free movement and an indication of daily life behaviour in the home environment of the target group. This set-up is a major advance in home assessment of mobility registration technology, allowing semi-structured validation and evaluation of a device after laboratory circumstances have been tested and following recently published recommendations [37]. The assessment is suitable for non-frail as well as frail older adults, since the standardized assessment is short and the free movement is tailored to a person’s capabilities.

A study limitation is found in the free movement. Subjects are instructed to perform daily tasks at will during thirty minutes. This instruction results in subjects performing several tasks in an often rushed manner, which they would normally be performing over a longer time span. This heightened activity causes accuracy in the free movement to be probably underrated. The moderate results of the detection of lying serve to illustrate this point. Another limitation is found in the nature of the tasks in the standardized assessment and free movement. While being fairly complete regarding postures, gait and indoor activities, bicycling was not included in the protocol. Since bicycling is a common activity among the very healthy older adult population, this could be included in further validation of the sensor.

## Conclusions

The necklace-worn sensor-based method is an acceptable valid and feasible instrument for detecting daily physical activity based upon ToL in frail and non-frail older adults. Accuracy for detecting ToL in daily activity is excellent. User acceptance of the sensor is high.

## Supporting Information

S1 Data Spreadsheet dataset. (XLSX)
